# Brain Training Game Boosts Executive Functions, Working Memory and Processing Speed in the Young Adults: A Randomized Controlled Trial

**DOI:** 10.1371/journal.pone.0055518

**Published:** 2013-02-06

**Authors:** Rui Nouchi, Yasuyuki Taki, Hikaru Takeuchi, Hiroshi Hashizume, Takayuki Nozawa, Toshimune Kambara, Atsushi Sekiguchi, Carlos Makoto Miyauchi, Yuka Kotozaki, Haruka Nouchi, Ryuta Kawashima

**Affiliations:** 1 Smart Ageing International Research Centre, Institute of Development, Aging and Cancer, Tohoku University, Sendai, Japan; 2 Japanese Society for the Promotion of Science, Tokyo, Japan; 3 Division of Developmental Cognitive Neuroscience, Institute of Development, Aging and Cancer, Tohoku University, Sendai, Japan; 4 Department of Functional Brain Imaging, Institute of Development, Aging and Cancer, Tohoku University, Sendai, Japan; 5 Institute for International Advanced Research and Education (IIARE), Tohoku University, Sendai, Japan; University of Pittsburgh, United States of America

## Abstract

**Background:**

Do brain training games work? The beneficial effects of brain training games are expected to transfer to other cognitive functions. Yet in all honesty, beneficial transfer effects of the commercial brain training games in young adults have little scientific basis. Here we investigated the impact of the brain training game (*Brain Age*) on a wide range of cognitive functions in young adults.

**Methods:**

We conducted a double-blind (de facto masking) randomized controlled trial using a popular brain training game (*Brain Age*) and a popular puzzle game (*Tetris*). Thirty-two volunteers were recruited through an advertisement in the local newspaper and randomly assigned to either of two game groups (*Brain Age*, *Tetris*). Participants in both the *Brain Age* and the *Tetris* groups played their game for about 15 minutes per day, at least 5 days per week, for 4 weeks. Measures of the cognitive functions were conducted before and after training. Measures of the cognitive functions fell into eight categories (fluid intelligence, executive function, working memory, short-term memory, attention, processing speed, visual ability, and reading ability).

**Results and Discussion:**

Our results showed that commercial brain training game improves executive functions, working memory, and processing speed in young adults. Moreover, the popular puzzle game can engender improvement attention and visuo-spatial ability compared to playing the brain training game. The present study showed the scientific evidence which the brain training game had the beneficial effects on cognitive functions (executive functions, working memory and processing speed) in the healthy young adults.

**Conclusions:**

Our results do not indicate that everyone should play brain training games. However, the commercial brain training game might be a simple and convenient means to improve some cognitive functions. We believe that our findings are highly relevant to applications in educational and clinical fields.

**Trial Registration:**

UMIN Clinical Trial Registry 000005618.

## Introduction

Can video game training, specifically that using commercial brain-training games, improve cognitive function in healthy young adults? Cognitive functions change throughout life. Some cognitive functions such as executive functions and working memory reach a peak in 20′s or 30′s [1]. Other cognitive functions such as semantic knowledge develop to the age of 60 or 70 [2]. Most cognitive functions of young adults at around 20 years of age do not reach the peak [3], [4], [5], [6]. Thus, improvements of cognitive functions by cognitive training in younger adults as well as in older adults are attracting attention. Video game training is cognitive training of one type [7], [8]. Video game training has attracted much attention because some video game training shows that effects of playing certain types of games have led to improvement of performances of other untrained tasks, which is commonly designated as a transfer effect [7], [9], [10], [11], [12]. The transfer effect was defined as “the ability to extend what has been learned in one context to new contexts” [13]. In the research fields of cognitive training using video games, the improvements of cognitive functions through playing video games were referred to as transfer effects [7], [8], [11], [12], [14], [15], [16]. In line with these results, commercial brain training games of many types (e.g., *Brain Age*, *Big Brain Academy*, and *Brain Challenge*) have been released. Such commercial brain training games have become popular around the world. The beneficial effects of these brain training games are expected to improve cognitive functions. However, a recent massive internet-based research of adults aged 18–60 demonstrated that brain training games of a certain type had no transfer effect on any other cognitive function [17]. For young adults, the beneficial effects of the commercial brain training games have little scientific basis. Investigation of transfer effects from commercial brain training games on cognitive functions is just beginning [8], [14], [15], [16].

Recent studies show that the effects of playing commercial brain training games such as *Brain Age*, published by Nintendo Co. Ltd., can lead improvement in the accuracy and speed of calculations in healthy children [15], [16] and can improve executive functions and processing speed in healthy elderly people [8], [14]. These results demonstrated that commercial brain training games, especially *Brain Age*, have a transfer effect in healthy children and healthy elderly people. However, it remains unclear whether the effects of playing the commercial brain training game transfer to cognitive functions in healthy young adults. Previous studies have showed that younger adults have a great possibility of improvement of cognitive functions through performing cognitive training and playing video games compared to older adults [9], [18], [19], [20], [21], [22]. These earlier results indicate that the effects of playing the brain training game can transfer to the cognitive functions in young adults.

It should be noted that cognitive performances improved after playing classic and recent videogames. For instance, playing the classic video games such as *Tetris*, Donkey Kong and Pac Man improved the reaction times [23], [24]. Moreover, playing the recent action video games such as Medal of Honor improved visuo-spatial and attentional skills [11], [12]. Thus, to prove the beneficial effects of the brain training game, it should necessary to compare improvement of cognitive functions after playing brain training games with that after playing other types of video games. In the present study, we selected *Brain Age* as the brain training game and *Tetris* as other types of video games for the reasons that these games were famous and easy to play. The further reasons why we used *Brain Age* and *Tetris* were described more fully in the method section.

This study investigated the beneficial transfer effects of a commercial brain training game on cognitive functions in the healthy young adults. To examine this issue, we conducted a de facto (double-blinded) intervention [25] with two parallel groups (a brain training group and an active control group). The de facto intervention [25] was a kind of double-blinded intervention which participants and testers were kept blind to the experimental hypothesis. The participants were asked to perform each type of video game training (*Brain Age* or *Tetris*) over 4 weeks with at least 5 training days in each week. On each training day, participants used the video game for about 15 min. This procedure was identical to that used in a previous intervention study of elderly people [8].

To evaluate the transfer effects of the commercial brain training game on cognitive functions, we assessed a broad range of cognitive functions (fluid intelligence, executive functions, working memory, short-term memory, attention, processing speed, visuo-spatial ability, and reading ability). Fluid intelligence was measured using Raven’s Advanced Progressive Matrices Test (RAPMT) [26]. Executive functions were measured using Wisconsin Card Sorting Test (WCST) [27], and Stroop Task (ST) [28]. Working memory was measured using Operation Span (OpS) [29], letter–number sequence (LNS) [30], and arithmetic (Ari) [30]. Short-term memory was measured using Digit Span (DS) [30] and Spatial Span (SpS) [31]. Attention was measured using the Digit Cancellation Task (D-CAT) [32] and Simple Reaction Time (SRT) [33]. Processing speed was measured using Digit Symbol Coding (Cd) [30] and Symbol Search (SS) [30]. Visuo-spatial ability was measured using the Mental Rotation task (MR) [34]. Reading (verbal) ability was measured using the Japanese Reading Test (JART) [35].

Based on previous studies [8], [14], [15], [16], [23], [24], we expected that playing video games would improve cognitive functions and the beneficial effects of video games on cognitive functions would differ according to the types of video games (*Brain Age* and *Tetris*). Moreover, we made three specific hypotheses related to improvements of cognitive functions after playing video games. First, playing *Brain Age* would lead to improve executive functions and processing speed compared with playing *Tetris*. The reason was that the previous study using *Brain Age* in the older adults showed improvements of executive functions and processing speed [8]. Second, playing *Brain Age* would improve working memory. Previous study showed that working memory was highly correlated with executive functions and processing speeds [36], [37]. Because playing *Brain Age* improved executive functions and processing speed [8], playing *Brain Age* would improve working memory in the same way. Thirdly, playing *Tetris* would improve the visuo-spatial ability and the attention compared with plying *Brain Age*. The previous study using *Tetris* demonstrated the improvements of the visuo-spatial ability and attention through playing *Tetris* [23], [24] [38].

## Materials and Methods

### Ethics Statement

In accordance with the Declaration of Helsinki, written informed consent was obtained from each subject. This study was approved by the Ethics Committee of the Tohoku University Graduate School of Medicine.

### Randomized Controlled Trial Design

This study was registered in the UMIN Clinical Trial Registry (UMIN 000005618). This randomized controlled trial was conducted between June 2011 and August 2011 in Sendai city, Miyagi prefecture, Japan. Written informed consent to participate in the study was obtained from each participant. The protocols for this study and supporting CONSORT checklist are available as supporting information (Checklist S1, Protocol S1 and Protocol S2).

To assess the impact of the brain training game on the young adults, we used a double-blind (de fact masking) intervention. Participants and testers were blind to the study’s hypothesis. Participants were blind to the treatment and control designations of these two groups, and were informed only that the study was designed to investigate the effects of two training programs. Testers were blind to the group membership of participants.

The researcher (N.R.) randomly assigned participants to either of two groups (*Brain Age*, *Tetris*) by a random draw using a computer in the following way. Participants were stratified by gender and randomized to the *Brain Age* or the *Tetris* groups by a random draw using the computer. In order to obtain a similar size of the *Brain Age* and the *Tetris* groups, blocked randomization (block size; 4) is applied with an allocation ratio of 1∶1.

### Participants

**Figure 1:**
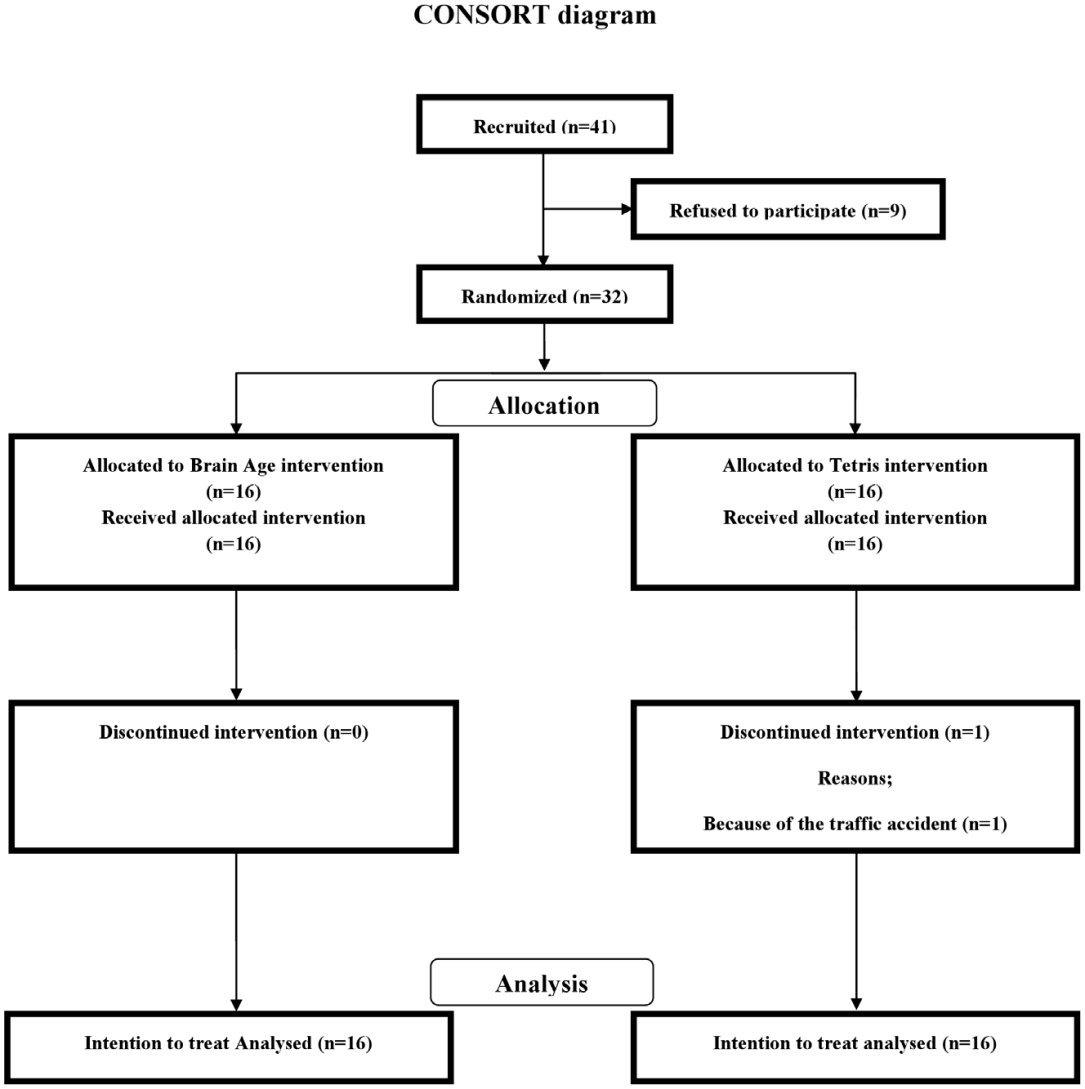
CONSORT diagram.

Forty-one participants, who were recruited through advertisements at a university and in the local newspaper, were screened using a questionnaire before inclusion (Figure 1). Nine participants declined to participate before a random assignment. All of the included participants (*n* = 32) reported being right-handed, were native Japanese speakers, were not concerned about their own memory functions, were not using medications known to interfere with cognitive functions (including benzodiazepines, antidepressants or other central nervous agents), and reported no disease known to affect the central nervous system, including thyroid disease, multiple sclerosis, Parkinson’s disease, stroke, severe hypertension, and diabetes. To maximize the benefit of the intervention, all participants were non-gamers and reported playing video games less than one hour of a week over the prior 2 years [11], [39].

All participants provided informed consent to participate in this study, which was approved by the Ethics Committee of the Tohoku University Graduate School of Medicine. After the written informed consent was obtained from each participant, participants were assigned randomly to either of two groups (*Brain Age*, *Tetris*) by a random draw using a computer (please see Randomized controlled trial design). The study was completed by 16 of the 16 members in the *Brain Age* group and 15 of the 16 members in the *Tetris* group (Figure 1). Table 1 presents the baseline demographic and neuropsychological characteristics of the participants included in the analyses. After the random assignment, we found no significant difference between the groups in terms of demographic or neuropsychological characteristics.

**Table 1:** Characteristics of participants in the *Brain Age* and *Tetris* groups.

	*Brain Age* group		*Tetris* group			
	(9M/7F)		(9M/7F)			
	Mean	SD	Mean	SD	Effect size (*d*)	*p*-value
Age (year)	20.50	1.10	20.87	1.25	0.31	0.36
Education (year)	13.50	1.10	13.80	1.05	0.28	0.43
JART (score)	20.56	3.79	19.00	2.90	0.46	0.22
RAPMT (score)	28.69	2.75	27.50	2.92	0.38	0.30

No significant difference was found between *Brain Age* and *Tetris* groups (two sample *t*-test, *p*>0.10). Effect size estimates were calculated using Cohen’s *d*. *d* = 0.20 is regarded as a small effect, *d = *0.50 as a medium effect, and *d = *0.80 as a large effect. M, number of men; F, number of women; JART, Japanese Reading test; RAPMT, Raven’s Advanced Progressive Matrices Test; SD, standard deviation.

### Overview of Intervention

The participants were asked to perform each video game training (*Brain Age* or *Tetris*) over 4 weeks with at least 5 training days each week. On each training day, participants performed the video game for about 15 min. We used *Brain Age* (Nintendo Co. Ltd.) as a game that participants in the brain training group played. We used *Tetris* (Nintendo) as a game that participants in the active control group played. The participants played video games on a portable console, Nintendo DSi (Nintendo Co. Ltd.), at home. The game performance was recorded for each participant. Nintendo DSi did not have functions to record the playing time and the playing date. Therefore, all participants were provided 1) a stopwatch which can record the playing time each training day and 2) a timer which informed the participants of the practice time (15 minutes). Participants used the stopwatch and the timer to keep to the practice time limit. At the end of each training day, participants reported the scores of the played games and the total practice time in a training diary. The *Brain Age* group listed the titles of trained games and a score for each trained game at the end of each training day. The *Tetris* group reported the best total score, total lines, and the final level at the end of each training day.

After the intervention period, we checked whether or not the practice time which participants reported in the training diary matched the actual practice time which were recorded in the stopwatch. Most practice times which participants reported were consistent with the actual practice times in the stopwatch. We used the actual practice time to estimate an average practice time. The average practice times in each training day were 15.85 minutes (SD = 1.49) in the *Brain Age* group and 15.92 minutes (SD = 1.37) in the *Tetris* group. There was no significant difference of the practice time between the *Brain Age* and the *Tetris* groups (*p*>0.1). This result suggested that the participants kept to the practice time (about 15minutes).

The measures of cognitive functions were conducted before and after training. On the first day of training (pre), all participants were tested on a series of neuropsychological and behavioral tests. After these tests, participants received instructions to play one of the games for 30 min. The following day, participants started 4 weeks of video game training. After 4 weeks of training (post), all participants were re-examined to assess their performance on some neuropsychological and behavioral tests. Finally, the portable console and the video game were returned at the end of the study.

We selected *Brain Age* because 1) *Brain Age* was one of the most popular brain training games in the world, 2) *Brain Age* was developed based on knowledge of neuroscience and psychological evidence [40], [41], and 3) results of previous studies indicated the beneficial effects of playing *Brain Age* on cognitive functions [8], [14], [15], [16]. Some previous studies have suggested that video game training studies should include an active control group that plays video games of other types [7], [39], [42]. Based on that suggestion, the active control group was designed to control for test–retest effects and positive effects to play some video games. We selected *Tetris* (Nintendo, 2006) as the active control game because 1) previous studies used *Tetris* as an active control group [8], 2) *Tetris* was one of the most popular video game, 3) previous study showed that participants can keep playing *Tetris* for 4 weeks [8], 4) playing *Tetris* improved spatial ability and attention and did not transfer broadly to other cognitive ability such as memory, executive functions [23], [24] [38].

#### Brain training group (Brain Age)

We used *Brain Age* (Nintendo Co. Ltd.) as a game which participants in the brain training group played. *Brain Age* is a popular brain training game developed based on previous findings of a cognitive training program for elderly people [8]. The previous study used reading aloud and simple arithmetic calculations as training tasks. There were some reasons why we selected these tasks. First, we hypothesized that activations of the association cortices by cognitive tasks may well improve regional cerebral blood flow and change brain activities and brain structures, which lead to improve the function of these cortices [10], [41], [43]. Second, we focused on the dorsolateral prefrontal cortex, because the dorsolateral prefrontal cortex is related to higher cognitive functions such as executive function, working memory and processing speed [44], [45], [46], [47], [48]. Moreover, these higher cognitive functions play a key role in the daily activity or in the social behaviors [49], [50], [51]. Third, these reading aloud and simple arithmetic calculations activated the dorsolateral prefrontal cortex activated in comparison to the resting state [52], [53]. Finally, these tasks were simple. Most games in *Brain Age* include elements of these reading aloud and simple arithmetic calculations.

*Brain Age* includes nine games. Based on a previous study [8], we used eight training games and avoided use of *Voice Calculation* because *Voice Calculation* is similar to *Calculation×20* and *Calculation×100*. 1) In *Calculation×20*, participants must answer 20 simple arithmetic calculations as quickly as possible. The questions include problems of mathematical addition, subtraction, and multiplication. 2) In *Calculation×100*, participants must answer 100 questions as quickly as possible. The questions include problems of mathematical addition, subtraction, and multiplication. 3) In *Reading Aloud*, participants must read aloud excerpts from Japanese classical literature. 4) In *Syllable Count*, some sentences written in a combination kanji and kana are presented. Participants must count the kana letters after translating kanji to kana. 5) In *Low to High*, numbers in boxes are first presented for a few seconds. Then participants must select the boxes from the lowest number to the highest number. 6) In *Head Count*, participants watch scenes in which some people enter or leave a house. Then participants must state the number of people in the house at the end. 7) In *Triangle Math*, three numbers are presented on a top line (e.g., 5, 7, 2), with two mathematical operations presented on a second line (e.g., +, +) and one mathematical operation (e.g. +) on the last line. First, participants must solve the first formula (5+7) using the first two numbers (5, 7) on the first line and the first mathematical operation (+) on the second line; then they must solve the second formula (7+2) using the last two numbers (7, 2) on the first line and the last mathematical operation (+) on the second line. Then, participants must solve the last formula using the answer of the first formula (12), the answer of the last formula (9), and the mathematical operation (+) on the last line. In this case, participants give the final answer (21). 8) In *Time Lapse*, two analog clocks are presented. Participants must calculate the difference in time between the two clocks. At the beginning of the game, participants can do only three training games (*Calculation×20*, *Calculation×100*, and *Reading Aloud*). New games are added to the game list after training for several days. After playing the games, the game performance at each game and the playing time of each game are recorded in the game memory (*Brain Age*). We used these actual game performances to confirm that playing the game actually improved the performance achieved when playing the trained games.

Participants received the following instructions. 1) Participants were asked to train for 15 minutes a day, five times a week during 4 weeks. 2) Participants were required to play the *Calculation×20*, the *Calculation×100*, and *Reading Aloud* games on each training day. 3) When a new training game was available, participants were allowed to play the new game. 4) Participants were restricted from playing the *Brain Age Check* because this game mode included a task that resembles the Stroop task, which is one measure of cognitive function. 5) After each training day, participants were required to check the name of the training games that had been played and to write down their high score of training in a training diary. Although the actual game scores were recorded in the game, to maintain motivation to participate in this study, we asked participants to write down the game performance data. After the intervention period, we checked whether or not the scores which participants reported matched the actual scores which were recorded in the video game. Although most scores which participants reported were consistent with the actual scores, we used the actual game scores from the game for our analyses.

#### Active control group (Tetris)

We used *Tetris* (Nintendo Co. Ltd.) as the game played by participants in the active control group. *Tetris* is a popular puzzle game in which players rotate and move blocks descending from the top of the screen so that these blocks form lines at the bottom of the screen. After a complete line with no gaps is formed, the line disappears. If no lines are formed, then the blocks pile higher and higher until the block pile reaches the top of the screen, at which point the game ends and the player loses. The goal is to keep the game going as long as possible by forming complete lines with the descending blocks. As the game progresses, the blocks descend faster, giving players less time to choose where to place each block. After playing the game, the game performance (total score) is recorded in the video game (*Tetris*). We used the actual game performance data to confirm that playing the game improved the trained game performance.

Participants received the following instructions. 1) Participants were asked to train for 15 minutes a day, five times a week during the 4 weeks. 2) After each training day, participants were required to write down the highest score they achieved in a training diary. Although the actual game scores were recorded in the game, to maintain motivation to participate in this study, we asked participants to write down the game performance data. After the intervention period, we checked whether or not the scores which participants reported matched the actual scores which were recorded in the video game. Although most scores that participants reported were consistent with the actual scores, we used the actual game scores from the game for our analyses.

### Overview of Cognitive Function Measures

To evaluate the transfer effects of the brain training game, we assessed a broad range of the cognitive functions. Fluid intelligence was measured using RAPMT [26], Executive functions were measured using WCST [27] and ST [28]. Working memory was measured using OpS [29], LNS [30], and Ari [30]. Short-term memory was measured using DS [30] and SpS [31]. Attention was measured using D-CAT [32] and SRT [33]. Processing speed was measured using Cd [30] and SS [30]. Visuo-spatial ability was measured using MR [34]. Reading (verbal) ability was measured using JART [35]. The primary outcome measure was ST [28]. We selected ST as the primary outcome measure because 1) *Brain Age* is expected to improve executive functions, 2) ST is an often-used task to measure executive functions [54], and 3) the procedure and score of ST have been standardized [28], [55].

#### RAPMT

Raven’s Advanced Progressive Matrices Test (RAPMT) measures fluid intelligence, including reasoning [26]. This test presents participants with a complex visual pattern with a piece cut out of it. The task of the participant is to find the missing piece that completes the pattern. RAPMT are published in two sets. Set I contains 12 diagrammatic puzzles, each with a missing part that one must attempt to identify from several alternatives. It is typically used for practice and to reduce anxiety. Participants practiced Set I before taking Set II. Set II has 36 puzzles that are identical in presentation to those in the practice set. The problems are presented in a bold, accurately drawn, and pleasant looking format to maintain interest and to minimize fatigue. In accordance with manual guidelines, a time limit of 30 min was given for completing the Set II. The primary measure for this task was the number of correct items.

#### WCST

Wisconsin Card Sorting Test (WCST) assesses executive functions including cognitive flexibility in response to feedback [27]. We used the PC version WCST with the developed Psychology Experiment Building Language (PEBL) test battery (http://pebl.sourceforge.net/) [27]. The total trial number is 128. Participants were required to sort the cards on the basis of color, shape, or number of figures. The only feedback provided to the subject was whether responses were correct or incorrect. The rule (color, shape, or number) was able to be switched as quickly as every tenth trial. The primary measure of this task was perseverative errors. The perseverative error was defined as an incorrect response to a shifted or new category that would have been correct for the immediately preceding category. The perseverative error is the most commonly used measure of WCST.

#### ST

Stroop task (ST) measures executive functions including response inhibition and impulsivity. We used Hakoda’s version Stroop test [28], [55]. Hakoda's version is a paper and pencil version of the Stroop test. In this test, participants must check whether their chosen answers are correct, unlike the traditional oral naming Stroop task. We used a reverse Stroop task (rST) and a Stroop task (ST). In the reverse Stroop task, in the leftmost of six columns, a word naming a color was printed in another color (e.g., “red” was printed in blue letters) and the other five columns were filled respectively, each with a different color, from which subjects had to check the column of which the color matched the written word in the leftmost column. In the Stroop task, in the leftmost of six columns, a word naming a color was printed in another color (e.g., “red” was printed in green letters) and the other five columns contained words naming colors. Subjects had to check the column containing the word naming the color of the word in the leftmost column. In each task, subjects were instructed to complete as many of these exercises as possible in 1 min. The primary measure for this task was the number of correct items.

#### OpS

Operation Span (OpS) measures working memory [29]. Participants solved math problems (e.g., IS (9/1) +2+9?) while simultaneously trying to remember sets of 3–6 words (this task is similar to Turner & Engle, 1989). After each set of 3–6 words, participants were asked to recall the words in the set in the order in which they were initially presented. Because this test was administered three times, three versions of the test were used. The primary measure of this task was accuracy of recall of word sets in the correct order.

#### LNS

Letter–Number Sequence (LNS) is a subtest in WAIS-III [30]. This test evaluates working memory. For this task, the examiner read a combination of letters and numbers; then participants were asked to recall numbers first in ascending order, followed by the letters in Japanese alphabetical order. If participants responded with letters first, followed by numbers, but with all of them in correct sequence, credit was awarded. LNS begins with the simplest level of a three-letter number sequence. There are five sets of the letters and numbers in increasing length, and each set consists of three trials (total 15 trials). The primary measures of this test are raw scores, which refer to the number of correctly repeated sequences. The maximum raw score is 15.

#### Ari

Arithmetic (Ari) is a subtest in WAIS-III [30]. This test evaluates working memory. In this task, the examiner reads arithmetic problems; then participants must solve the arithmetic problems without the use of a pencil and paper. This task is a mental arithmetic task. Ari has 21 arithmetic problems which is the same as problems in WAIS-III. The primary measure of this test is the raw score. The maximum row score is 21.

#### DS

Digit Span (DS) is a subtest in WAIS-III [30]. This test evaluates verbal short-term memory. Digit Span has two subsections (DS-F and DS-B). For DS-F, participants answer numbers in the same order as they were read aloud by the examiner. For DS-B, participants answer numbers in the reverse order of that presented aloud by the examiner. In both, the examiner reads a series of number sequences in which the examinee must answer the sequence in either forward or reverse order. DS-F and DS-B begin with simplest level of two digits in sequence. The number of digits in the sequence is increased one by one to a maximum number irrespective of participants’ answers. The maximum span length of DS-F is eight. The maximum span length of DS-B is seven. Each span length consists of two trials. The raw score of DS-F is 16. The raw score of DS-B is 14. The primary measures of these tests are raw scores.

#### SpS

Spatial Span (SpS) is a subtest in WMS-R [31]. This test evaluates visual short-term memory. The Spatial Span test has two subsections (SpS-F and SpS-B). In SpS, participants must memorize sequences of locations and orders presented on a screen. For each trial, eight squares are shown on the screen; then a sequence of black squares flashed yellow, each square changing color for 1000 ms with a 500 ms interval between squares. At the end of the sequence, participants answer the locations in the same order in which they are presented (SpS-F), and in the reverse order (SpS-B). SpS-F and SpS-B begin with simplest level of two squares in sequence. The number of squares in the sequence is increased one by one to a maximum number irrespective of participants’ answers. The maximum span length of SpS-F is seven. The maximum span length of SpS-B is six. Each span length consists of two trials. The raw score of SpS-F is 14. The raw score of SpS-B is 12. The primary measures of these tests are raw scores.

#### D-CAT

Digit Cancellation Task (D-CAT) evaluates attention [32]. The test sheet consists of 12 rows of 50 digits. Each row contains five sets of numbers 0–9 arranged in random order. Consequently, any one digit would appear five times in each row with randomly determined neighbors. D-CAT consists of three such sheets. Participants were instructed to search for the target number(s) that had been specified to them and to delete each one with a slash mark as quickly and as accurately as possible until the experimenter sent a stop signal. Three trials were used, first with a single target number (6), second with two target numbers (9 and 4), and third with three (8, 3, and 7). Each trial was given 1 minute. Consequently, the total time required for D-CAT was 3 min. In the second and third trials, it was emphasized that all instructed target numbers should be cancelled without omission. The primary measure of this test is the number of hits (correct answers). We used only the number of hits in the first trial.

#### SRT

Simple Reaction Time (SRT) evaluates attention [33]. In the SRT test, a single stimulus “X” appeared in the center of the screen. The participant was instructed to press the enter key with the right index finger as quickly as possible when the stimulus appeared. The stimulus reappeared with a random delay ranging from 250 ms to 2500 ms (250, 500, 750, 1000, 1250, 1500, 1750, 2000, 2250, and 2500). The test has 4 blocks of 50 trials. The total number of trials was 200 trials. The primary measure in this task is reaction time on the SRT. The reasons why we selected the SRT as an attentional measure are 1) previous study suggested the SRT can measure attention [33] [56], [57], [58], [59]. 2) Some cognitive assessment battery (Cambridge Neuropsychological Test Automated Battery; CANTAB (http://www.cantab.com/) and Clinical Assessment for Attention; CAT (http://shinkoh-igaku.jp/kigu/catcas.html)) classified the SRT as an attentional measure, 3) previous study using factor analysis demonstrated that the SRT was classified into the attentional measure factor [60] and 4) calculated Cronbach's alpha for attentional measure (D-CAT and SRT) using the data in the pre training. The Cronbach’s alpha of attentional measure was 0.67. A commonly accepted Cronbach’s alpha is over 0.7, although a value of 0.6 can be accepted during exploratory research [61], [62]. Thus, we considered the SRT as an attentional measure in the present study.

#### Cd

Symbol Coding (Cd) is a subtest of WAIS-III [30]. This test measures processing speed. For Cd, the participants are shown a series of symbols that are paired with numbers. Using a key, the participants draw each symbol under its corresponding number within a 120 s time limit. The primary measure of this test is the number of correct answers.

#### SS

Symbol search (SS) is a subtest of WAIS-III [30]. This test measures processing speed. The SS contains 60 items. For this subtest, the participants visually scan two groups of symbols (a target group and a search group) and indicate if either of the target symbols matches any of the symbols in the search group. The participants respond to as many items as possible within a 120 s time limit. The primary measure of this test is the number of correct answers.

#### MR

Mental rotation (MR) measures visuo-spatial ability [34]. Participants try to determine whether two simultaneously presented shapes are the same or different. They responded as quickly and as accurately as possible by pressing one of two keys. The shape on the right was either the same shape or a mirror image of the shape on the left, and the two shapes differed in orientation by 0°, 45°, 90°, 135°, 180°, 225°, 270°, or 315°. All other shapes can appear equally often at each rotation from 0° to 315°. Participants completed 10 practice trials followed by 128 test trials. The primary measure in this task is the average reaction time in each rotation from 0° to 315° on the mental rotation task. Analyses include only trials in which a correct response was made by the participants.

#### JART

Japanese Reading Test (JART) measures reading ability [35]. JART is a Japanese version of the National Adult Reading Test (NART), which has a reading test of 50 irregularly spelled words in English (e.g. ache). JART is a reading test consisting of 25 Kanji compound words (e.g., ??, ??). The reading stimuli were printed out randomly for reading. The subjects were asked to read each Kanji compound word aloud. This task assesses reading ability and IQ. The primary measure for this task is the number of correct items.

### Questionnaires of Subjective Feeling for the Intervention

Previous study suggested that differences of subject feeling (e.g. motivation, fatigue, satisfactions) between intervention groups may affect improvement of cognitive functions [42]. Based on the suggestion, we asked participants to answer the questionnaires related to the subjective feelings (1; motivation of playing the video game during the intervention period, 2; fatigue during the intervention period, 3; satisfaction of the intervention during the intervention period, 4; enjoyment of the video game during the intervention period) after the intervention period. Participants rated these questionnaires using a nine-point scale (for motivation scale, from 1 = very low to 9 = very high; for fatigue scale, from 1 = very low to 9 = very high; for satisfaction scale, from 1 = very low to 9 = very high; for enjoyment scale, from 1 = very low to 9 = very high).

### Data Analysis

This study was conducted to evaluate the effects of the brain training game on cognitive functions. The pre- and post- training scores in cognitive functions were presented in Table 2. We calculated a change score (post-training score minus pre-training score) in all measures of cognitive functions (Table 3). We conducted the permutation tests of an analysis of covariance (ANCOVA) for the change scores in each of cognitive tests. The permutation test for ANCOVA was conducted using the function “aovp” of the Imperm package (http://cran.r-project.org/web/packages/lmPerm/index.html) in R version.2.14.1 (http://www.r-project.org/). The change scores were the dependent variable, groups (*Brain Age*, *Tetris*) was the independent variable. Pre-training scores in each cognitive test, sex, and age were the covariates to exclude the possibility that any pre-existing difference of measures between groups affected the results of each measure and to adjust for background characteristics. The level of significance was set at *p*<0.05.

**Table 2:** Cognitive function scores at before and after training in both groups.

	*Brain Age* Group					*Tetris* Group					
	Pre		Post			Pre		Post		Comparison of scores in pre-scores	
	Mean	SD		Mean	SD	Mean	SD	Mean	SD	Effect size (*d*)	*P*-value
Fluid intelligence											
RAPMT (score)	28.69	(2.75)		32.13	(2.90)	27.60	(2.92)	30.13	(2.96)	0.38	0.310
Executive functions											
WCST (percentage of error response)	13.69	(3.55)		11.31	(1.99)	14.60	(4.55)	14.27	(3.51)	0.22	0.550
rST (score)	61.81	(7.92)		68.19	(10.25)	60.27	(8.45)	60.80	(8.13)	0.19	0.610
ST (score)	49.31	(5.99)		53.13	(5.81)	49.20	(7.77)	50.00	(7.35)	0.02	0.960
Working memory											
OpS (percentage of correct response)	70.06	(17.29)		83.75	(9.06)	62.13	(20.01)	68.27	(16.30)	0.42	0.370
LNS (score)	10.50	(2.71)		12.81	(1.87)	10.07	(1.91)	10.60	(2.43)	0.18	0.620
Ari (score)	16.69	(1.35)		18.44	(1.59)	16.13	(2.30)	16.60	(2.47)	0.29	0.430
Short-term memory											
DS-F (score)	12.13	(2.50)		12.75	(2.24)	11.93	(1.73)	12.93	(1.43)	0.09	0.810
DS-B (score)	11.69	(2.09)		11.94	(1.57)	11.53	(1.93)	11.67	(2.19)	0.07	0.850
SpS-F (score)	10.50	(2.68)		10.81	(2.26)	10.27	(1.43)	10.47	(1.67)	0.11	0.770
SpS-B (score)	8.50	(2.00)		8.81	(2.14)	8.67	(2.98)	8.93	(2.23)	0.06	0.860
Attention											
D-CAT (number)	35.69	(7.29)		37.69	(6.05)	35.80	(7.21)	37.13	(7.64)	0.02	0.860
SRT (ms)	283.73	(32.83)		280.79	(24.09)	291.67	(24.67)	275.11	(20.04)	0.27	0.460
Processing speed											
Cd (number)	106.81	(9.13)		115.44	(9.65)	104.33	(13.07)	106.73	(11.85)	0.22	0.550
SS (number)	55.56	(3.24)		59.38	(2.99)	54.00	(6.25)	54.67	(6.95)	0.31	0.400
Reading ability											
JART (score)	20.56	(3.79)		20.81	(3.53)	19.00	(2.89)	19.60	(2.70)	0.46	0.220
Visuo-spatial ability											
MR (ms)	1711.5	(332.1)		1644.5	(358.4)	1714.6	(359.7)	1375.3	(314.3)	0.01	0.990

Group comparison (two sample *t*-tests) of the pre-training scores revealed no significant difference in any measure of cognitive functions between the brain training group and the *Tetris* training group (*p*>0.10). Effect size estimates were calculated using Cohen’s *d*. *d* = 0.20 is regarded as small effect, *d = *0.50 as medium effect, and *d = *0.80 as large effect. Pre, pre-training; post, post-training; SD, standard deviation. Fluid intelligence was measured using Raven’s Advanced Progressive Matrices Test (RAPMT). Executive functions were measured using Wisconsin Card Sorting Test (WCST) and Stroop Task (ST). Working memory was measured using the Operation Span (OpS), letter–number sequence (LNS), arithmetic (Ari). Short-term memory was measured using the Digit Span (DS) and Spatial Span (SpS). Attention was measured using the Digit Cancellation Task (D-CAT) and Simple Reaction Time (SRT). Processing speed was measured using the Digit Symbol Coding (Cd) and Symbol Search. Visuo-spatial ability was measured using the Mental Rotation task (MT). Reading (verbal) ability was measured using the Japanese Reading Test (JART).

**Table 3:** Change scores in cognitive functions measures of both groups.

	*Brain Age* Group		*Tetris* Group		Results of ANCOVAs		Results of Additional ANCOVAs	
	Mean	SD	Mean	SD	Effect size (*η^2^*)	*P*-value	Effect size (*η^2^*)	*P*-value
Fluid intelligence								
RAPMT (score)	3.44	(1.93)	2.53	(1.86)	0.08	0.126	0.07	0.210
Executive functions								
WCST (percentage of error response)	−2.38	(2.53)	−0.33	(1.62)	0.23	0.000	0.24	0.000
rST (score)	6.38	(8.24)	0.53	(5.10)	0.20	0.007	0.19	0.007
ST (score)	3.81	(3.41)	0.80	(1.76)	0.25	0.002	0.25	0.005
Working memory								
OpS (percentage of correct response)	0.15	(0.18)	0.06	(0.14)	0.15	0.003	0.15	0.006
LNS (score)	2.31	(2.24)	0.53	(1.82)	0.14	0.005	0.14	0.005
Ari (score)	1.75	(1.29)	0.47	(1.50)	0.21	0.008	0.13	0.013
Short-term memory								
DS-F (score)	0.63	(1.45)	1.00	(1.93)	0.00	0.626	0.00	0.765
DS-B (score)	0.25	(1.65)	0.13	(1.67)	0.00	0.923	0.00	0.960
SpS-F (score)	0.31	(2.39)	0.20	(1.87)	0.00	0.554	0.00	0.633
SpS-B (score)	0.31	(1.58)	0.27	(1.34)	0.00	0.863	0.00	0.941
Attention								
D-CAT (number)	2.00	(4.83)	1.33	(4.70)	0.00	0.690	0.00	0.921
SRT (ms)	−2.94	(18.42)	−16.56	(15.30)	0.10	0.012	0.10	0.012
Processing speed								
Cd (number)	8.63	(5.44)	2.40	(6.24)	0.23	0.006	0.24	0.003
SS (number)	3.81	(3.67)	0.67	(3.03)	0.24	0.004	0.24	0.005
Reading ability								
JART (score)	0.25	(1.00)	0.60	(1.50)	0.00	0.961	0.00	0.980
Visuo-spatial ability								
MR (ms)	−66.95	(243.82)	−339.39	(271.25)	0.18	0.003	0.19	0.005

Change scores were calculated by subtracting the pre-cognitive measure score from the post-cognitive measure score. We conducted the two types of permutation tests of an analysis of covariance (ANCOVA) for the change scores in each of cognitive tests. In the first ANCOVA, pre-training scores in each cognitive test, sex, and age were the covariates. The results were presented in lines of results of ANCOVAs. In the second ANCOVA, pre-training scores in each cognitive test, sex, age and pre-training score of SRT were the covariates. The results were presented in lines of results of additional ANCOVAs. The level of significance was set at *p*<0.05.

Moreover, we report eta square (η2) as an index of effect size. As a descriptive index of strength of association between an experimental factor (main effect or interaction effect) and a dependent variable, η2 is defined as the proportion of total variation attributable to the factor, and it ranges in value from 0 to 1. η2≥0.01 is regarded as a small effect, η2≥0.06 as a medium effect, and η2≥0.14 as a large effect.

SD, standard deviation. Fluid intelligence was measured using Raven’s Advanced Progressive Matrices Test (RAPMT). Executive functions were measured using Wisconsin Card Sorting Test (WCST) and Stroop Task (ST). Working memory was measured using the Operation Span (OpS), letter–number sequence (LNS), arithmetic (Ari). Short-term memory was measured using the Digit Span (DS) and Spatial Span (SpS). Attention was measured using the Digit Cancellation Task (D-CAT) and Simple Reaction Time (SRT). Processing speed was measured using the Digit Symbol Coding (Cd) and Symbol Search. Visuo-spatial ability was measured using the Mental Rotation task (MT). Reading (verbal) ability was measured using the Japanese Reading Test (JART).

There were some reasons why we used the permutation tests for ANCOVA models. First, the permutation test is a suitable for small sample analysis and is distribution free [63], [64], [65], [66]. Second, the permutation test can correct Type 1 error (false positive) [67], [68]. There are some methods (e.g. multiple testing corrections and re-sampling) to control Type 1 error. Bonferroni [69] and Benjamini and Hochberg (False discovery rate; FDR) [70] correction methods are typical multiple testing correction methods. Permutation test is a typical resampling method [71]. The Bonferroni correction is known to be extremely conservative. It can lead to Type II (i.e. false negative) errors of unacceptable levels, which may contribute to publication bias and the exclusion of potentially relevant hypotheses [72]. In contrast, FDR method is less stringent, which may lead to the selection of more false positives. Thus, permutation tests have become widely accepted and recommended in studies that involved multiple statistical testing [67], [68], [72].

Moreover, we report eta square (*η^2^*) as an index of effect size. As a descriptive index of strength of association between an experimental factor (main effect or interaction effect) and a dependent variable, *η^2^* is defined as the proportion of total variation attributable to the factor, and it ranges in value from 0 to 1 [73]. Using information (the sums of squares for total; SS total, the sum of squares for factor; SS factor) from the ANCOVAs, we calculate *η^2^* (SS factor divided by SS total). SS factor is the variation attributable to the factor and SS total is the total variation which includes the SS factor and the sum of squares for error. In actuality, *η^2^*≥0.01 is regarded as a small effect, *η^2^*≥0.06 as a medium effect, and *η^2^*≥0.14 as a large effect [73]. Group comparison (two sample *t*-tests) of the pre-training scores revealed no significant difference in any measure of cognitive function between the *Brain Age* group and the *Tetris* group (*p*>0.10, Table 2).

We also conducted two sample *t*-tests for questionnaires of the subjective feeling (motivation, fatigue, satisfaction, enjoyment). The level of significance was set at *p*<0.05. Moreover, Effect size estimates were calculated using Cohen's *d* [73]. *d* = 0.20 is regarded as a small effect, *d = *0.50 as a medium effect, and *d = *0.80 as a large effect.

Missing data were imputed using Missing Value Analysis in the Statistical Package for the Social Sciences (SPSS). In particular, we imputed missing values using maximum likelihood estimation based on the expectation–maximization algorism with the observed data in an iterative process [74]. All randomized participants were included in the analyses in line with their allocation (intention-to-treat principle).

### Sample Size

Our sample size estimation was based on the change score in rST (please see cognitive function measures). The sample size was determined using a calculation developed by Borm [75] for two-group ANCOVA (*Brain Age* vs. *Tetris*) in the context of randomized trials. A previous study showed the average score (57.29) and standard deviation (7.59) of rST in the young adults (age 20–29) [55]. The correlation of rST between subsequent 4 week periods was *r* = 0.751. We expected to detect a difference of 5 change score in rST between *Brain Age* and *Tetris* Group. The sample size calculation indicated that the sample size of approximately 16 would achieve a power of 0.80 using two-tailed tests with an alpha of 0.05.

## Results

As presented in Figure 1, the 32 participants in this study were randomized into two groups (*Brain Age* and *Tetris*). The study was completed by 16 of the 16 members in the *Brain Age* group and 15 of the 16 members in the *Tetris* group. Table 1 presents the baseline demographics and neuropsychological characteristics of the participants. Based on intention to treat principle, we imputed missing values of one participant in the *Tetris* group (see Data Analysis). Before analyzing the transfer effects of the brain training game to other cognitive functions, we examined whether the practice improved the performances of the trained games. Participants in both groups showed significant improvement of game performance achieved during the last time playing compared to the first time playing (paired *t*-test, *p*<0.05, Table 4).

**Table 4:** First and last game scores in both *Brain Age* and *Tetris* training groups.

			Pre	Post	Effect size (*d* )	*p*-value	Training days	Maximum training days
*Tetris* training group								
	Total score (score)	M	106369.30	355423.67	1.38	0.00	24.87	28
		SD	(117697.42)	(225860.30)			(2.53)	
	Total line (number)	M	95.00	193.53	1.87	0.00		
		SD	(60.28)	(60.04)				
	Final Level (level)	M	10.07	19.67	1.86	0.00		
		SD	(5.92)	(5.99)				
*Brain Age* training group								
	*Calculation×20* (s)	M	19.25	14.75	0.70	0.04	25.12	28
		SD	(9.23)	(3.17)			(1.96)	
	*Calculation×100* (s)	M	113.50	84.19	1.96	0.00	24.68	28
		SD	(31.84)	(14.92)			(2.30)	
	*Reading Aloud* (word/min)	M	8.16	10.38	0.63	0.04	25.06	28
		SD	(1.76)	(4.27)			(2.02)	
	*Low to High* (score)	M	40.56	45.69	0.59	0.03	24.81	27
		SD	(6.56)	(9.13)			(1.97)	
	*Syllable Count* (s)	M	139.00	104.19	0.71	0.01	23.75	26
		SD	(49.12)	(37.02)			(2.54)	
	*Head Count* (score)	M	4.44	4.94	0.56	0.04	22.38	25
		SD	(0.63)	(0.68)			(2.96)	
	*Triangle Math* (s)	M	45.63	34.56	0.58	0.04	12.50	15
		SD	(20.47)	(9.27)			(2.50)	
	*Time Lapse* (s)	M	105.94	73.81	0.99	0.00	10.88	13
		SD	(38.81)	(19.17)			(2.45)	

Significant differences were found between first and last game scores in both *Brain Age* and *Tetris* (paired *t*-test, *p*<0.05). Effect size estimates were calculated using Cohen's *d*. *d* = 0.20 is regarded as a small effect, *d = *0.50 as medium effect, and *d = *0.80 as large effect. In the *Brain Age* group, the maximum number of training days on each training game was different because the training games were added to the training list through training. Pre, pre-training; post, post-training; M, mean; SD, standard deviation.

To evaluate the transfer effect of the brain training game on the improvement of other cognitive functions, we conducted ANCOVA for the change scores in each of the cognitive tests (Table 3). Results showed that the *Brain Age* group improved all measures of the executive functions (WCST, *F* (1, 27) = 20.28, *η^2^* = 0.23, *p* = 0.000; ST, *F* (1, 27) = 10.09, *η^2^* = 0.25, *p* = 0.002; rST, *F* (1, 27) = 8.22, *η^2^* = 0.20, *p* = 0.007), all measures of the working memory (OpS, *F* (1, 27) = 9.48, *η^2^* = 0.15, *p* = 0.003; LNS, *F* (1, 27) = 9.19, *η^2^* = 0.14, *p* = 0.005; Ari, *F* (1, 27) = 7.43, *η^2^* = 0.21, *p* = 0.008), and all measures of the processing speed (Cd, *F* (1, 27) = 9.46, *η^2^* = 0.23, *p* = 0.006; SS, *F* (1, 27) = 9.65, *η^2^* = 0.24, *p* = 0.004) compared to the *Tetris* group. These results demonstrate that the effects of playing *Brain Age* were transferred to executive functions, working memory, and processing speed. Because the training games in *Brain Age* required participants to response as quickly as possible, there was a possibility that the performance of SRT would affect the results of improvements of cognitive functions. To check the possibility, we conducted the additional analyses using the SRT score before playing the video games as a covariate. The results were the similar to the results which did not use the SRT score as a covariate. These results represented in Table 3. These results indicated that the reaction time did not affect the improvements of cognitive functions.

On the other hand, the *Tetris* group improved one of two measures of attention (SRT, *F* (1, 27) = 6.08, *η^2^* = 0.10, *p* = 0.012) and the measure of visuo-spatial ability (MR, *F* (1, 27) = 8.42, *η^2^* = 0.18, *p* = 0.009) compared to the *Brain Age* group. These results show that the effects of playing *Tetris* were transferred to attention and visuo-spatial ability.

However, playing *Brain Age* or *Tetris* did not improve one the measure of fluid intelligence (RAPMT, *F* (1, 27) = 3.09, *η^2^* = 0.08, *p* = 0.126), one of two measures of attention (D-CAT, *F* (1, 27) = 0.07, *η^2^* = 0.00, *p* = 0.690), any measure of short-term memory (DS-F, *F* (1, 27) = 0.26, *η^2^* = 0.00, *p* = 0.626; DS-B, *F* (1, 27) = 0.01, *η^2^* = 0.00, *p* = 0.923; SpS-F, *F* (1, 27) = 0.20, *η^2^* = 0.00, *p* = 0.554; SpS-B, *F* (1, 27) = 0.00, *η^2^* = 0.00, *p* = 0.863), or the measure of reading ability (JART; *F* (1, 27) = 0.01, *η^2^* = 0.00, *p* = 0.918).

To investigate the differences of the subject feelings (e.g. motivation) between the groups, we conducted two samples t-test for questionnaires of the subjective feelings. There were no significant differences of the subjective feeling (Table 5).

**Table 5:** Subjective feeling for intervention in the *Brain Age* and *Tetris* groups.

	*Brain Age* group		*Tetris* group			
	(9M/7F)		(9M/7F)			
	Mean	SD	Mean	SD	Effect size (*d*)	*p*-value
Motivation	6.25	1.43	6.33	1.45	0.05	0.87
Fatigue	3.43	1.63	3.53	1.35	0.06	0.86
Satisfaction	6.56	1.82	6.46	2.06	0.05	0.89
Enjoyment	6.51	2.26	6.40	2.36	0.05	0.78

No significant difference was found between *Brain Age* and *Tetris* groups (two sample *t*-test, *p*>0.10). Effect size estimates were calculated using Cohen's *d*. *d* = 0.20 is regarded as a small effect, *d = *0.50 as a medium effect, and *d = *0.80 as a large effect. M, number of men; F, number of women; Motivation, motivation of playing the video game during the intervention period; Fatigue, fatigue during the intervention period; Satisfaction, satisfaction of the intervention during the intervention period; Enjoyment, enjoyment of the video game during the intervention period; SD, standard deviation.

## Discussion

The most important findings of this study were that playing the commercial brain training game (*Brain Age*) significantly improved executive functions, working memory, and processing speed compared to playing the non-brain training game (*Tetris*) in young adults. The present results demonstrated the beneficial transfer effects of the commercial brain training game on widely various cognitive functions in young adults. Moreover, these results showed that playing *Tetris* can engender improvement attention and visuo-spatial ability compared to playing *Brain Age*. These findings are consistent with previous evidence showing that playing video games can engender improvement in untrained cognitive functions [7], [8], [11], [12], [42].

In cognitive training studies, the transfer effect can be classified also in terms of a near transfer effect and a far transfer effect [76], [77]. The near transfer effect refers to improvements in cognitive domains that are closely related to the trained cognitive processes. In contrast, the far transfer effect refers to improvements in cognitive domain that are not closely related to the trained cognitive processes. From the viewpoints of the near and far transfer effects, the cognitive measures in this study are divisible into two measures of the transfer effects (near and far). For the *Brain Age* playing group, executive functions, working memory, and processing speed were the measures of the near transfer effect. The others were measures of the far transfer effects. The reason is that the training domains of *Brain Age* would be expected to train executive functions, working memory, and processing speed. For the *Tetris* playing group, attention and visuo-spatial ability were the measures of the near transfer effect; the others were the measures of the far transfer effects because the training of *Tetris* would be expected to train attention and visuo-spatial ability. Our results show that playing *Brain Age* and *Tetris* had only the near transfer effects, but not the far transfer effects. Some explanations might be applicable for the absence of the far transfer effect in this study. First, the possibility exists that the training term of our study (4 weeks) is not a sufficient time to obtain the far transfer effect. Second, our video game training was not intended for use as an adaptive training method. Results of previous studies suggest that the adaptive training method is more effective for improvement of cognitive functions than a non-adaptive training program [43], [78]. Because *Brain Age* and *Tetris* did not change the difficulty of tasks depending on the participant’s performance, we were unable to identify the far transfer effect.

The mechanism of the near transfer effects through playing *Brain Age* can be explained using a recent hypothesis, which proposes that the transfer effect can be induced if the processes during both training and transfer tasks are overlapped and are involved in similar brain regions [8], [19]. Most training games in *Brain Age* entail an element of the calculations and readings [8]. To perform these processes, the prefrontal regions [52], [53] or the precuneus [79], [80] should be recruited. The executive functions, working memory, and processing speed, which showed a significant transfer effect by the brain training game in this study, also involve the prefrontal cortex [81], [82], [83] and the precuneus [84], [85], [86], [87], [88]. These findings suggest that both training games and transfer tasks can share the same brain region, the prefrontal cortex or the precuneus, and that the near transfer effects of the brain training game on the executive functions, working memory, and processing speed can be mediated by the prefrontal regions or the precuneus. Further studies will be necessary to test this hypothesis using neuroimaging techniques (e.g. magnetic resonance imaging; MRI or magnetoencephalography; MEG). Brain activations or brain structures (e.g. gray matter volume or white matter integrity) in these regions may be changed after playing video games.

To test the overlapped hypothesis using the data of the present study, we conducted Spearman’s rank correlation analyses between a change score of training games in *Brain Age* (a last game score minus a first game score) and the change score of cognitive functions. The analyses showed that 1) the change score of *Calculation×20* significantly correlated with the change score of Ari (*Spearman's ρ* = .54, *p*<.05) and that of ST (*Spearman's ρ* = .51, *p*<.05), 2) the change score of *Calculation×100* significantly correlated with the change score of ST (*Spearman's ρ* = .56, *p*<.05), and 3) *Reading aloud* significantly correlated with the change score of Ari (*Spearman's ρ* = .66, *p*<.05). There were not significant correlations between the change score of other training games and the change score of cognitive functions. These results partially support the overlapped hypothesis. The reason of lacks of the correlation may be because of the number of participants (*N* = 16) is apparently not suitable to identify the relationships between the change score of other training games and the change score of cognitive functions. To confirm the overlapped hypothesis, further studies using a larger sample size should be needed.

The current study presents several strengths compared with earlier studies [8], [14], [15], [16], [17], [89] that have investigated the beneficial effects of brain training games on cognitive functions. First, unlike previous studies [14], [89], we used a randomized controlled trial with an active control group (*Tetris* group). The randomized controlled trial is an excellent means to provide effectiveness of cognitive training. Using the active control group is expected to control for test–retest effects and positive effects to play some video games. Therefore, our study can provide sufficient scientific evidence of the beneficial effects of the commercial brain training games on cognitive functions. Secondly, in contrast to many other studies [8], [14], [15], [16], [17], [89], we measured widely various cognitive functions. Therefore, results show that the brain training game had the near transfer effect, but not the far transfer effect.

It is important to consider why the previous study [90] could not show the improvement of cognitive functions after playing other types of brain training game for 20 hours. There were some disadvantages in the previous study [90]. First, the previous study used Big Brain Academy as the brain training game. Unlike in the case of *Brain Age*, the video game did not base on the scientific evidences. Secondly, the previous study used a within-participants (crossover) procedure, where each participant took part in both the brain training session and the reading articles session (control condition). Although this crossover design can reduce individual differences and sample size, the design has some limitations. The most significant problem of the crossover design is the ‘carryover’ effect. The carryover effect is defined as the persistence (whether physically or in terms of effect) of treatment (intervention) applied in one treatment phase of the study to subsequent treatment (intervention) phases. Thirdly, the previous study reported that most of the participants were not excited about playing Big Brain Academy after the study [90]. On the other hand, our participants felt satisfaction and enjoyment for playing *Brain Age*. Because of these disadvantages, the previous study [90] did not find the improvement of cognitive functions after playing Big Brain Academy for 20 hours.

It is essential to discuss the similar and the different results between the previous study using *Brain Age* and *Tetris* in the older adults [8] and the present study in the younger adults. The similar result was that *Brain Age* improved the executive functions and processing speed in both the younger adults and the older adults. These results would prove the efficacy of playing *Brain Age* on cognitive functions for the healthy adults regardless of age. The different result was that the present results in the young adults showed the improvements of working memory after playing *Brain Age* and the improvements of visuo-spatial ability and attention which was measured SRT after playing *Tetris*. Previous study [8] did not measure working memory, visuo-spatial ability and attention using SRT. Thus, it is not clear yet that *Brain Age* can improve working memory in the older adults and *Tetris* can improve visuo-spatial and attention measured SRT in the older adults. Additional research should be needed to investigate using the same cognitive measures.

It is also important to consider the limitations of this study. First, we did not assess the long-term benefit of playing the brain training game. Some previous studies showed that cognitive training had a long-term benefit on cognitive functions [89], [91]. One important future direction is to examine whether or not the brain training game can be expected to have long-term beneficial effects on cognitive functions. Second, the brain training game (*Brain Age*) consisted of multiple training games (see the Methods section). Multiple training programs can demonstrate intense transfer effects [42], [92]. However, it would be difficult to identify the beneficial effects of each training program on cognitive functions [10]. Third, we did not use the multiple cognitive measures for the visuo-spatial ability. Previous studies [12], [38] demonstrated the relationships between experiences of playing video games and the visuo-spatial ability. Because we use only the mental rotation task as the visuo-spatial ability, it is still unclear about the beneficial effects of the brain training game on the other types of visuo-spatial ability. It would be importance to consider whether or not the brain training game can improve a wide range of visuo-spatial ability. Fourth, we measured the subject feelings (motivation, fatigue, satisfactions) only once (after intervention). The subject feeling would change (increase or decrease) during the intervention period. To investigate the influence of the subjective feeling on improvements of the cognitive functions in more detail, further study which will measure the subjective feelings several times during an intervention period will be necessary. Fifth, there is a possibility that a difference between types of video game would affect the improvements of the cognitive functions. For example, *Brain Age* can act as a kind of a virtual coach who encourages systematically the participants to improve their performances. On the other hand, *Tetris* did not systematically facilitate the improvement of the performance. In the present study, the motivation and the satisfaction after playing video games did not differ between *Brain Age* and *Tetris*. However, the difference of types of video games may influence the improvement of the cognitive functions. A future study would be needed to investigate the effect of the virtual coach of brain training games on the cognitive functions.

To conclude, this study produced scientific evidence demonstrating that the commercial brain training game had beneficial effects on cognitive functions (executive functions, working memory, and processing speed) in healthy young adults. Our previous study, which used the same intervention method, also demonstrated that playing the brain training game improved executive functions and processing speed in healthy elderly people [8]. Results of the present and previous studies demonstrate that the commercial brain training game can at least improve executive functions and processing speed in healthy young people and healthy older adults. Our results do not indicate that everyone should play brain training games because 1) we did not compare the beneficial effects of brain training games to the beneficial effect of other effective training methods (e.g., fitness training or working memory training) [10], [93], 2) the brain training game had only the near transfer effect on cognitive functions [8], and 3) some limitations are applicable, as discussed above. However, the commercial brain training game might be a simple and convenient means to improve some cognitive functions. We believe that our findings are highly relevant to applications in educational and clinical fields. Some reports of previous studies have described that that the brain training game can support classroom activities for children [15], [16] and that they can improve cognitive functions (e.g. executive functions) for older adults [8], [14]. One important future direction is to examine whether or not the brain training game can support educational and clinical activities.

## Supporting Information

Checklist S1 **CONSORT Checklist.**
(DOC)

Protocol S1 **Trial Protocol.**
(PDF)

Protocol S2 **Trial Protocol (Japanese version).**
(PDF)
