# Supplementary material for: Brain Training Game Boosts Executive Functions, Working Memory and Processing Speed in the Young Adults: A Randomized Controlled Trial
Source: PLoS One. 2013 Feb 6;8(2):e55518. doi: 10.1371/journal.pone.0055518 (PMC3566110; doi:10.1371/journal.pone.0055518)
Supplement: Protocol S1 — Trial Protocol. (PDF) [file pone.0055518.s002.pdf]

The efficacy of playing the Nintendo DS games on cognitive functions in the healthy young adults: a randomized controlled trial.

Trial Protocol

**Title**	The efficacy of playing the Nintendo DS games on cognitive functions in the healthy young adults: a randomized controlled trial.
**Summary**	The aim of this trial is to investigate the effect of brain training games intervention (15 minutes per day, at least 5 days per week, for 4 weeks) on the cognitive functions (executive function, working memory, short-term memory, attention, processing speed, visual ability, and reading ability).
**Study Design**	Randomized controlled trial<br>Double -blind (participants and tester blinded)
**Description**	Since the brain training games were first released, they have been extremely popular around the world. The beneficial effects of these brain training games are expected to improve to other cognitive functions, which is commonly referred to as a transfer effect. Yet in all honesty, the beneficial transfer effects of the brain training games have little scientific basis.<br><br>**Purpose**<br>To reveal the impact of the brain training game on cognitive functions in the healthy young adults, we plan to conduct a double-blinded randomized controlled trial using Brain Age which is one of the popular brain training game.<br><br>**Participants**<br>Thirty-two participants are recruited through an advertisement in the local newspaper and screened by a questionnaire before inclusion. All participants are right-handed, native Japanese speakers, not concerned about their own memory functions, not using medications known to interfere with cognitive functions (including benzodiazepines, antidepressants or other central nervous agents), and having no diseases known to affect the central nervous system, including thyroid disease, multiple sclerosis, Parkinson's disease, stroke, severe hypertension or diabetes.<br>To maximize the benefit of the intervention, all participants are non-gamers and reported playing less than one hour of video games a week over the past 2 years.<br><br>**Overview of intervention**<br>The participants are asked to perform each video game training (Brain Age or Tetris) over 4 weeks with 5 training days in each week. On each training day, participants perform the video game for about 15 minutes. The participants play video games on the portable console, Nintendo DSi, at their homes. Game performance is recorded for each participant. At the end of each training day, participants report the scores of the played games. The Brain Age group lists the titles of trained games and a score for each trained game at the end of each training day. The Tetris group only reports the best total score at end of each training day. The measures of cognitive functions are conducted before and after training. On the first day of training (pre), all

	participants are tested on a series of neuropsychological and behavioral tests. After these tests, participants receive the instruction to play one of the games for 30 minutes. The following day, participants start 4 weeks video game training. After 4 weeks of training (post), all participants are re-examined on some neuropsychological and behavioral tests.<br>The procedures for this study have been approved by the Ethics Committee of the Tohoku University Graduate School of Medicine.
**Primary Outcome Measure(s)**	To evaluate the transfer effects of the brain training game, we assessed a broad range of the cognitive functions (executive function, working memory, short-term memory, attention, processing speed, visuo-spatial ability, and verbal ability).<br>Executive functions was measured by 1)Raven's Advanced Progressive Matrices Test (RAPMT), Wisconsin Card Sorting Test (WCST) and 2)Stroop Task (ST). Working memory was measured by 3)Operation Span (OpS), 4)letter-number sequence (LNS), 5)arithmetic (Ari). Short-term memory was measure by 6)Digit Span (DS) and 7)Spatial Span (SpS). Attention was measured by 8)Digit Cancellation Task (D-CAT) and 9)Simple Reaction Time (SRT). Processing speed was measure by 10)Digit Symbol Coding (Cd) and 11)Symbol Search(SS). Visuo-spatial ability was measured by 12)Mental Rotation task (MT). Reading (verbal) ability was measured by 13)Japanese Reading Test (JART).
**Secondary Outcome Measure(s)**	1)Brain structure measured by MRI
**Sample Size**	Our sample size estimation was based on the change score in rST. The sample size was determined using a calculation developed by Borm et al. [4] for 2-group ANCOVA (Brain Age vs Tetris) in the context of randomized trials. Previous study showed that average score (57.29) and standard deviation (7.59) of rST in young adults (age 20-29) (the manual of Hakoda's version Stroop task). The correlation of rST between subsequent 4 week periods was r = .751. We expected to detect a different of 5 change score in rST between Brain Age and Tetris Group. The sample size calculation indicated that the sample size of approximately 16 would achieve a power of .80 using 2-tailed tests at an alpha of .05.
**Setting**	Sendai city, Miyagi prefecture in Japan
**Project Coordinator**	Rui Nouchi (Ph.D)<br>Researcher,<br>Institute of Development, Aging and Cancer, Tohoku University, Smart Aging International Research Center, Seiryo-machi 4-1, Aoba-ku Sendai
**Overall Study Official(s)**	Institute of Development, Aging and Cancer, Tohoku University, Smart Aging International Research Center, Seiryo-machi 4-1, Aoba-ku Sendai
**Human Subjects Review/Oversight**	This research was approved by the Ethics Committee of the Tohoku University Graduate School of Medicine.
**Collaborators**	Ryuta Kawashima[1,2,3], Yasuyuki Taki[2], Hikaru Takeuchi[1], Hiroshi Hashizume[2],

	Takayuki Nozawa[1], Atsushi Sekiguchi[3], Yuka Kotozaki[1], Kambara Toshimune[3,4], Makoto, C. Miyauchi[3], Nouchi Haruka[1]<br><br>1 Smart Ageing International Research Centre, Institute of Development, Aging and Cancer, Tohoku University, Sendai 980-8575, Japan<br>2 Division of Developmental Cognitive Neuroscience, Institute of Development, Aging and Cancer, Tohoku University, Sendai 980-8575, Japan<br>3 Department of Functional Brain Imaging, Institute of Development, Aging and Cancer, Tohoku University, Sendai 980-8575, Japan<br>4 Japanese Society for the Promotion of Science, Tokyo102-8472, Japan
**Funding Source**	The Research Institute of Science and Technology for Society/ Japan Science and Technology Agency
